# Supplementary material for: Endocytosis and Digestion in Carnivorous Pitcher Plants of the Family Sarraceniaceae
Source: Plants (Basel). 2019 Sep 24;8(10):367. doi: 10.3390/plants8100367 (PMC6843295; doi:10.3390/plants8100367)
Supplement: Supplementary file 1 [file plants-08-00367-s001.pdf]

plants

Supplementary Figures

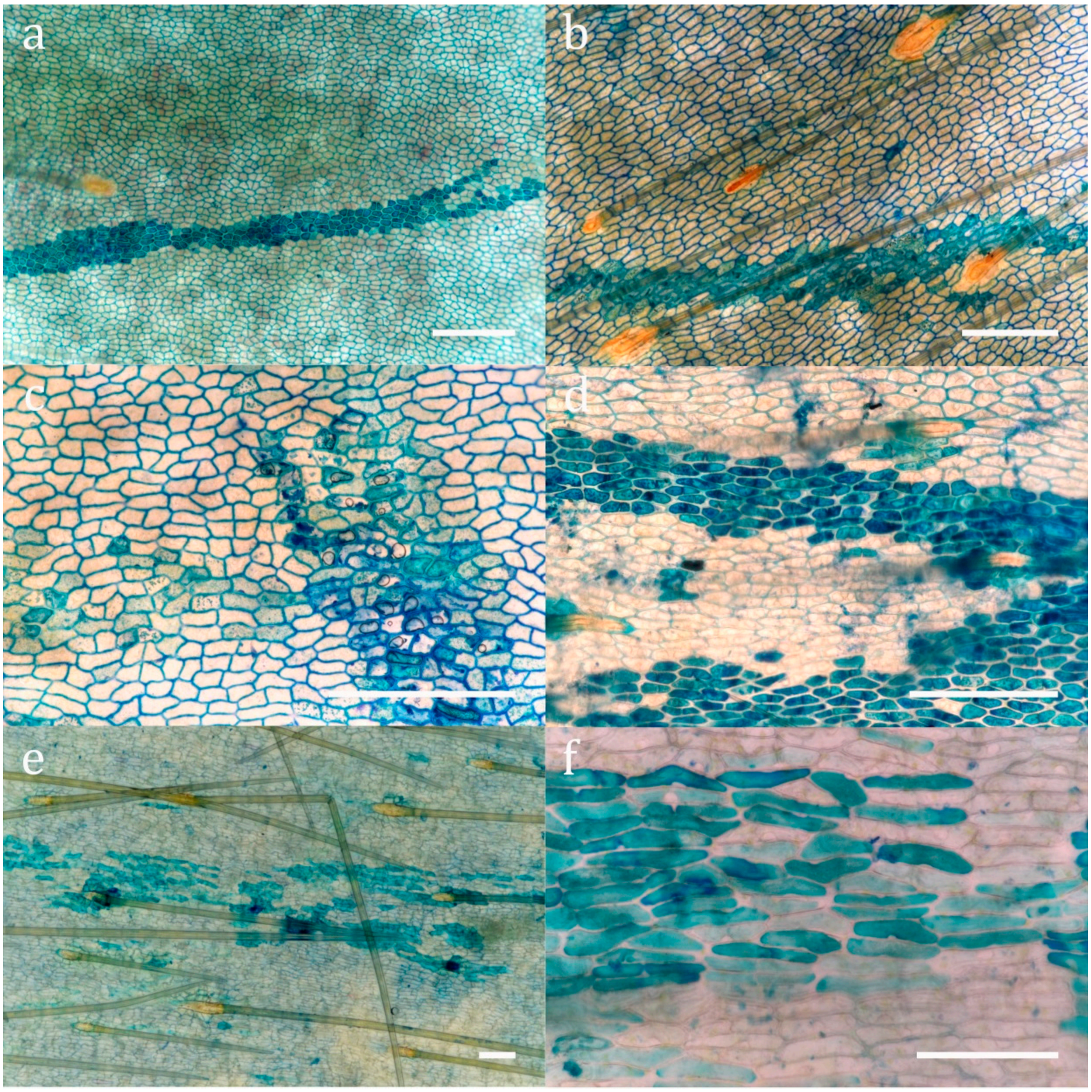

Supplementary Figure S1. Methylene blue staining of cuticular pores in the absorptive zone of the other tested species (a) *S. purpurea* ssp. *purpurea* (b) *S. purpurea* ssp. *venosa* (c) *S. rosea* (d) *S. flava* (e) *Heliamphora nutans* - overview (f) *Heliamphora nutans* - detail. Scale: 100 µm.

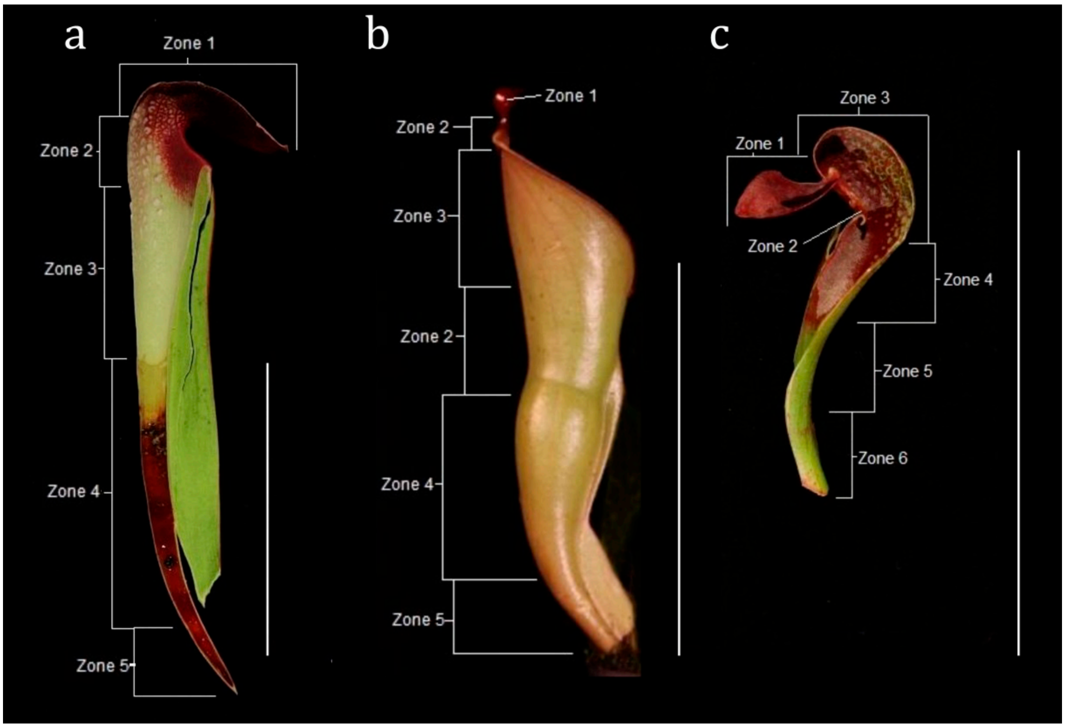

Supplementary Figure S2. Absorptive zones of Sarraceniaceae according to the terminology of Juniper et al. [20]: (a) *Sarracenia minor*: Zone 4; (b) *Heliamphora nutans*: Zone 4; (c) *Darlingtonia californica*: Zone 5. Vertical scale: 10 cm.

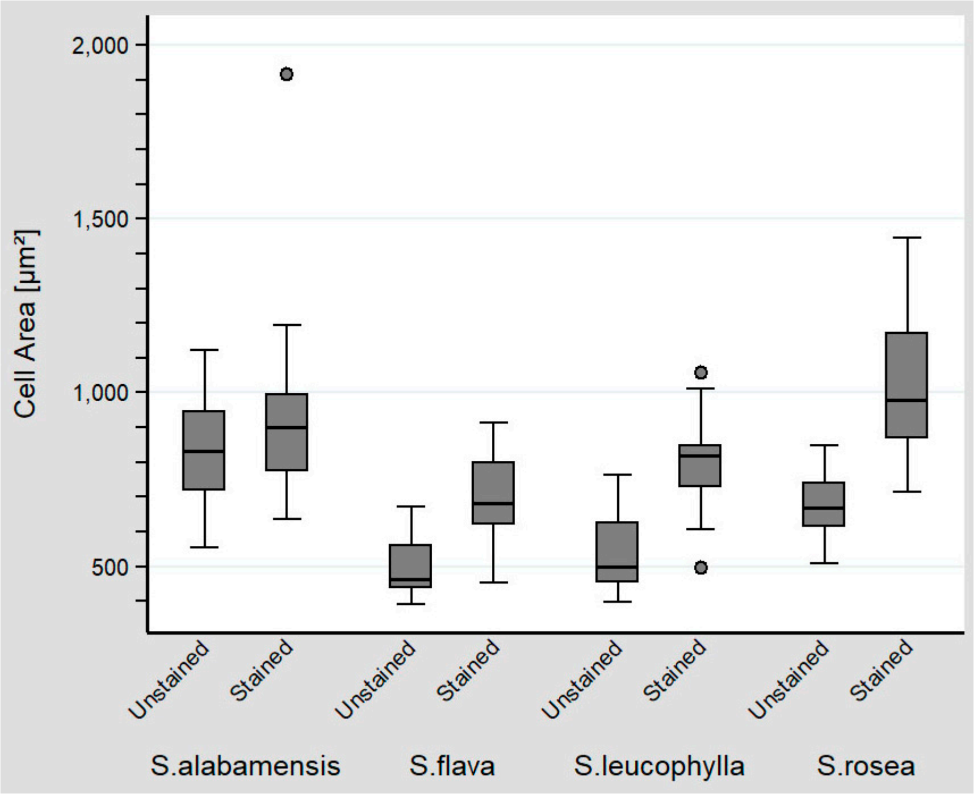

Supplementary Figure S3. Cell area of permeable and impermeable cells in 4 species of *Sarracenia*. Differences are highly significant ($p<0.001$) for *S. flava*, *S. leucophylla* and *S. rosea* but not significant for *S. alabamensis* ($p=0.211$).
